# Clinical Implication of Inflammation-Based Prognostic Score in Pancreatic Cancer: Glasgow Prognostic Score Is the Most Reliable Parameter

**DOI:** 10.1097/MD.0000000000003582

**Published:** 2016-05-06

**Authors:** Suguru Yamada, Tsutomu Fujii, Norimitsu Yabusaki, Kenta Murotani, Naoki Iwata, Mitsuro Kanda, Chie Tanaka, Goro Nakayama, Hiroyuki Sugimoto, Masahiko Koike, Michitaka Fujiwara, Yasuhiro Kodera

**Affiliations:** From the Department of Gastroenterological Surgery (Surgery II), Nagoya University Graduate School of Medicine (SY, TF, NY, NI, MK, CT, GN, HS, MK, MF, YK), and Center for Advanced Medicine and Clinical Research (KM), Nagoya University Hospital, Aichi, Japan.

## Abstract

A variety of systemic inflammation-based prognostic scores have been explored; however, there has been no study to clarify which score could best reflect survival in resected pancreatic cancer patients.

Between 2002 and 2014, 379 consecutive patients who underwent curative resection of pancreatic cancer were enrolled. The Glasgow Prognostic Score (GPS), modified GPS (mGPS), neutrophil to lymphocyte ratio (NLR), platelet to lymphocyte ratio (PLR), prognostic index (PI), and prognostic nutritional index (PNI) scores for each patient were calculated. Survival of each score was evaluated, and correlations between the score selected on the basis of the prognostic significance and various clinicopathological factors were analyzed.

In the analysis of the GPS, the median survival time (MST) was 28.1 months for score 0, 25.6 for score 1, and 17.0 for score 2. As for mGPS, the MST was 25.8 months for score 0, 27.7 for score 1, and 17.0 for score 2. Both scores were found to be significant. On the contrary, there were no statistical differences in MST between various scores obtained using the NLR, PLR, PI, or PNI. Multivariate analysis revealed that lymph node metastasis, positive peritoneal washing cytology, and a GPS score of 2 were significant prognostic factors. There was also statistically significant correlation between the GPS score and tumor location (head), tumor size (≥2.0 cm), bile duct invasion, and duodenal invasion.

Our study demonstrated that the GPS could be an independent predictive marker and was superior to other inflammation-based prognostic scores in patients with resected pancreatic cancer.

## Abstract 2

Supplemental Digital Content is available in the text

## INTRODUCTION

Pancreatic cancer continues to have the worst prognosis of all gastrointestinal malignancies, with complete surgical resection offering the only possibility of a cure. A number of clinicopathological factors, such as lymph node status,^1^ tumor size,^2^ portal vein invasion,^3^ surgical margin,^4^ curative resection,^5^ and adjuvant chemotherapy,^6^ have been advocated as prognostic indicators in patients with resected pancreatic cancer. On the contrary, it has been recognized that tumor progression and outcome are influenced by a variety of host-related factors. In particular, some studies have shown that infiltration of tumor microenvironment by inflammatory cells plays an important role in tumor development and progression.^7–9^ Furthermore, chronic inflammation represents both an important etiologic factor in the development of pancreatic cancer and a reactionary process to pancreatic cancer.^10^ Therefore, it is important to understand the biological mechanisms that contribute to tumor development and progression and to identify host-related prognostic factors.

In recent years, the prognostic significance of a variety of systemic inflammation-based prognostic scores has been explored in different cancers such as lung, esophageal, colorectal, and renal.^11–15^ Among these prognostic scores, Glasgow Prognostic Score (GPS) based on serum C-reactive protein (CRP) and albumin, neutrophil to lymphocyte ratio (NLR), platelet to lymphocyte ratio (PLR), prognostic index (PI) based on CRP and white blood cell count, and prognostic nutritional index (PNI) based on albumin and lymphocyte count are recognized as useful in predicting outcomes after surgery in regard to host-related factors.^16^ However, there has been no study to clarify which inflammation-based prognostic score could best reflect survival in a large cohort of resected pancreatic ductal adenocarcinoma patients.

The aim of the current study was to evaluate the clinical value of various inflammation-based prognostic scores as predictors in our large cohort of patients who underwent curative resection of pancreatic cancer and identify the most promising scoring system and to statistically analyze the correlation between the identified inflammation-based prognostic score and clinicopathological factors.

## METHODS

### Patient Selection

Between April 2002 and December 2014, 379 consecutive patients who underwent curative resection of pancreatic cancer at the Department of Gastroenterological Surgery (Surgery II), Nagoya University Graduate School of Medicine, were enrolled in this study. The Ethics Committee of the hospital approved the study and informed consent was obtained from all patients for the subsequent use of their resected tissues. In our institution, endoscopic retrograde cholangiopancreatography (ERCP) and stent implantation have been routinely performed in the patients with jaundice before surgery. All of the patients in this study underwent pancreatic resection after the preoperative serum bilirubin level was normalized before surgery. The median interval between the biliary drainage and surgery was 29 days. A mesenteric approach and a nontouch isolation technique were used, and extended radical lymph node dissection (D2) with paraaortic lymph node sampling was performed on all patients with no macroscopically apparent liver or peritoneal metastases.^17,18^ Patients were followed for a median of 15.1 months (range, 0.43–150.7 months) or until death.

Resected pancreatic tumors were pathologically confirmed to be invasive ductal adenocarcinomas. Patients with intraductal papillary mucinous neoplasms, endocrine tumors, or other cystic tumors were excluded from this study. The TNM staging system for pancreatic tumors of the Union for International Cancer Control (UICC; seventh edition) was used.^19^ The following tumor characteristics were examined microscopically postoperatively: differentiation, invasion of the anterior pancreatic capsule or retroperitoneal tissue, plexus invasion, resection margin, and lymph node metastasis. The main pancreatic duct of the remnant pancreas was pathologically examined by frozen section, and negative surgical margins were confirmed during surgery.

Adjuvant chemotherapy comprised gemcitabine and/or S-1, and the oral 5-fluorouracil prodrug tegafur with oteracil and gimeracil. Unless contraindicated by the patient's condition or rejection, adjuvant chemotherapy was administered to all patients. Gemcitabine (1000 mg/m^2^) was administered weekly for 3 weeks followed by 1 week of rest. Oral S-1 was administered from days 1 to 14, followed by a 1-week rest period. Chemotherapy was started within 2 months of surgery in all patients who were considered eligible for this treatment.

### Inflammation-Based Prognostic Score

Preoperative blood samples were drawn 1 or 2 days before surgery. Serum CRP and albumin concentrations were measured (HITACHI, LABOSPECT 008), as were white blood cells, neutrophils, lymphocytes and platelets (HITACHI, Sysmex XN-9000). The GPS and modified GPS (mGPS), NLR, PLR, PI, and PNI scores for each patient were calculated as described in Supplementary Table. Cut-off values for the NLR, PLR, and PNI were determined on the basis of previous studies.^16^

### Statistical Analysis

Overall survival (OS) was defined as the time from surgery to death from any cause. OS curves according to the GPS, mGPS, NLR, PLR, PI, and PNI were constructed using the Kaplan–Meier method and compared using the log-rank test. Factors significant on univariate analysis were entered into the multivariate Cox proportional hazards model, and the hazard ratio (HR) and its 95% confidence interval (CI) were calculated. Correlations between the GPS score and clinicopathological factors were analyzed statistically. Differences in the numerical data between the 2 groups were evaluated using Fisher exact test or χ^2^ test. Data were analyzed using JMP version 10 software (JMP; SAS Institute, Cary, NC). The level of statistical significance was set at *P* < 0.05.

## RESULTS

### Patient Demographics

Table 1 summarizes the demographics of the 379 patients in this study. The mean age at presentation was 65.0 years [standard deviation (SD), 9.6 years]; there were 228 male and 151 female subjects. Tumors were located in the pancreatic head in 291, body and tail in 82, and entire organ in 6 patients. One hundred forty-one pancreatoduodenectomies, 28 pylorus-preserving pancreatoduodenectomies, 112 subtotal stomach-preserving pancreatoduodenectomies, 70 distal pancreatectomies, 26 total pancreatectomies, 1 medial pancreatectomy, and 1 pancreatic head resection with segmental duodenectomy were performed. The conclusive stages of the 379 patients who underwent resection according to the UICC classification^19^ were IA in 12 cases, IB in 2 cases, IIA in 110 cases, IIB in 217 cases, III in 2 cases, and IV in 35 cases (Table 1).

**TABLE 1:**
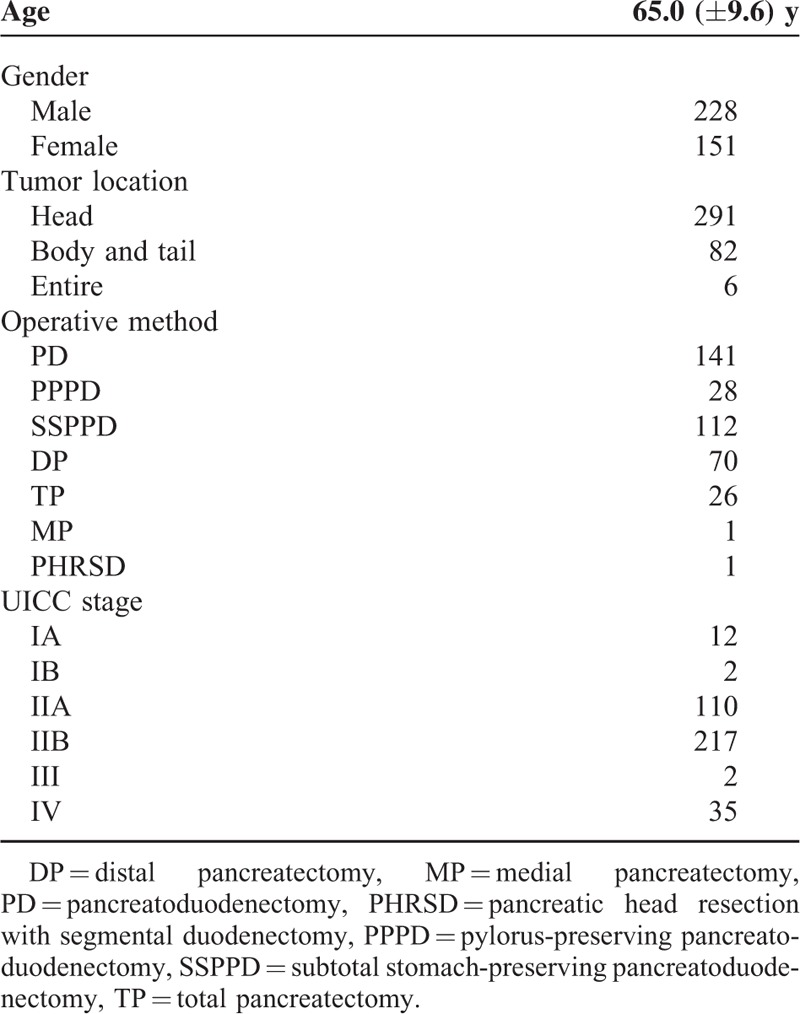
Patient Demographics

### OS Based on Inflammation-Based Prognostic Score

**FIGURE 1:**
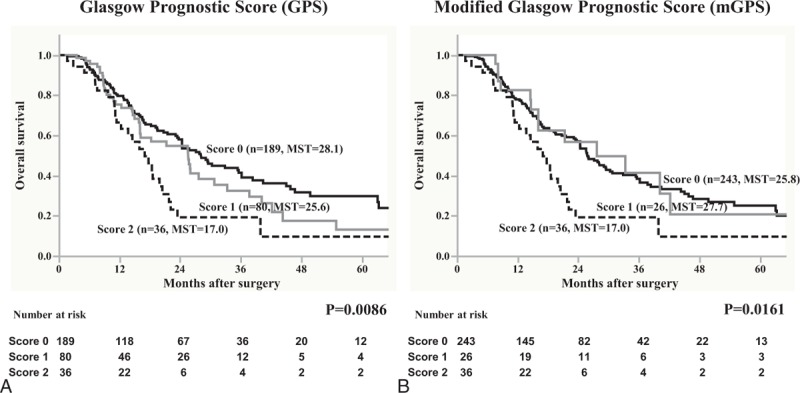
Overall survival in 379 patients with pancreatic cancer was statistically analyzed on the basis of (A) Glasgow Prognostic Score (GPS) and (B) modified Glasgow Prognostic Score (mGPS) as inflammation-based prognostic scores. MST = median survival time.

OS in the 379 patients with pancreatic cancer was assessed using 6 different inflammation-based prognostic scores. First, survival was analyzed using the GPS and mGPS. In the analysis of the GPS, the median survival time (MST) was 28.1 months for score 0, 25.6 for score 1, and 17.0 for score 2, which was statistically significant (*P* = 0.0086) (Figure 1A), whereas for mGPS, the MST was 25.8 months for score 0, 27.7 for score 1, and 17.0 for score 2, which was also statistically significant (*P* = 0.0161) (Figure 1B).

**FIGURE 2:**
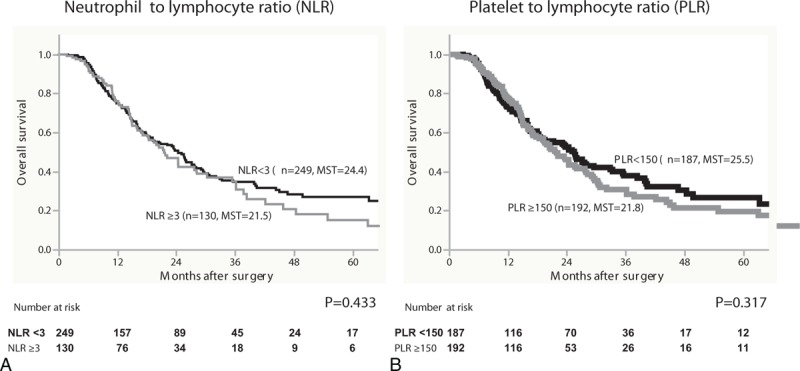
Overall survival in 379 patients with pancreatic cancer was statistically analyzed on the basis of (A) neutrophil to lymphocyte ratio (NLR) and (B) platelet to lymphocyte ratio (PLR) as inflammation-based prognostic scores. MST = median survival time.

Second, survival was evaluated using the NLR and PLR. As a result, when patients were divided using the cut-off level of NLR = 3, the MST for the NLR < 3 group was 24.4 months, whereas the MST for the NLR ≥3 group was 21.5, which was not statistically significant (Figure 2A). Similarly, when patients were divided using the cut-off level of PLR = 150, the MST for the PLR < 150 group was 25.5 months, whereas the MST for the PLR ≥150 group was 21.8, which was also not statistically significant (Figure 2B).

**FIGURE 3:**
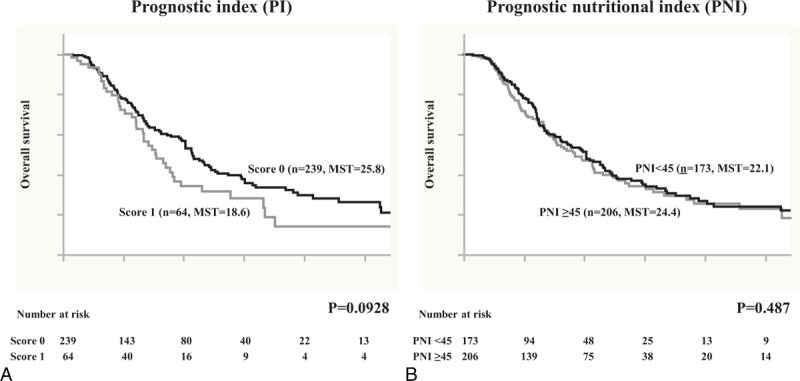
Overall survival in 379 patients with pancreatic cancer was statistically analyzed on the basis of (A) prognostic index (PI) and (B) prognostic nutritional index (PNI) as inflammation-based prognostic scores. MST = median survival time.

Finally, survival was evaluated on the basis of the PI and PNI. In the analysis of the PI, the MST in patients with score 0 was 25.8 months, whereas for those with score 1, it was 18.6 months. Although there was a trend toward a survival difference, it did not reach statistical significance (Figure 3A). However, the MST for patients in the PNI < 45 group was 22.1 months and for those in the PNI ≥45 groups was 24.4; it was not statistically significant (Figure 3B).

### Univariate and Multivariate Analysis of Clinicopathological Parameters

Among the 6 different inflammation-based prognostic scores, the GPS score was demonstrated to be the most promising predictive factor in resected pancreatic cancer patients. Therefore, multivariate analysis was conducted using various predictive factors, which were significant in univariate analysis, including the GPS score. As a result, multivariate analysis revealed that CEA (≥5 ng/mL) (HR: 1.556, *P* = 0.029), lymph node metastasis (HR: 1.842, *P* = 0.0067), positive peritoneal washing cytology (HR: 1.830, *P* = 0.019), and a GPS score of 2 (identical to mGPS score of 2) (HR: 1.723, *P* = 0.028) were significant independent prognostic factors in resected pancreatic cancer patients (Table 2).

**TABLE 2:**
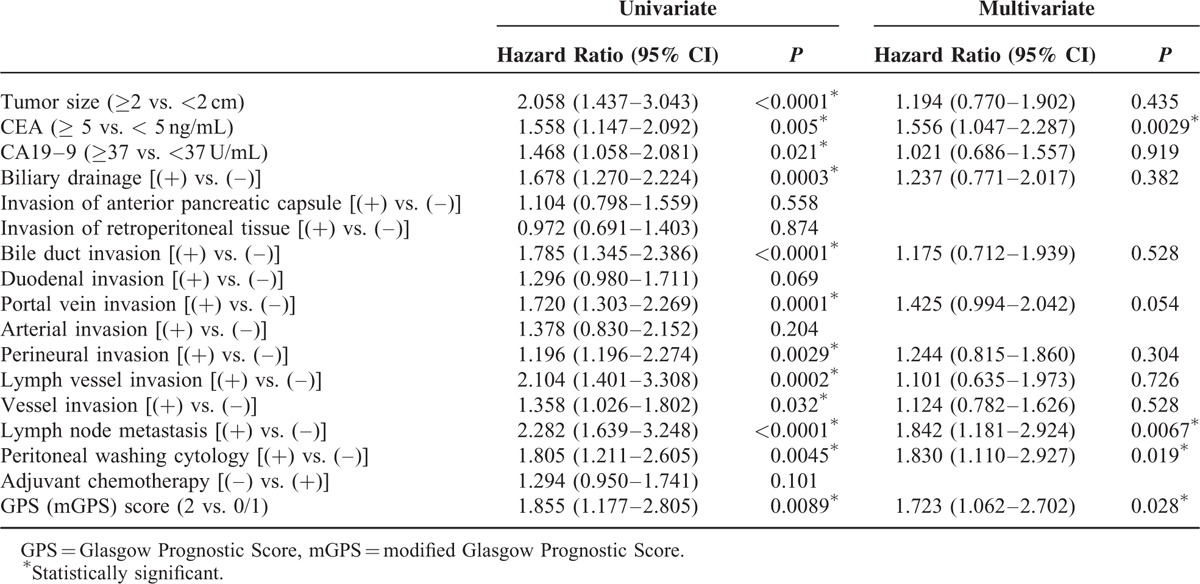
Univariate and Multivariate Analysis of Clinicopathological Parameters

### Correlation Between GPS Score and Clinicopathological Factors

To better understand the clinical implication of the GPS score, the correlation between the GPS score and various clinicopathological factors was statistically analyzed (Table 3). Increase in the GPS score and tumor location (head) (*P* = 0.0025), preoperative biliary drainage (*P* < 0.0001), tumor size (≥2.0 cm) (*P* = 0.0402), bile duct invasion (*P* < 0.0001), and duodenal invasion (*P* < 0.0001) were found to be statistically significant.

**TABLE 3:**
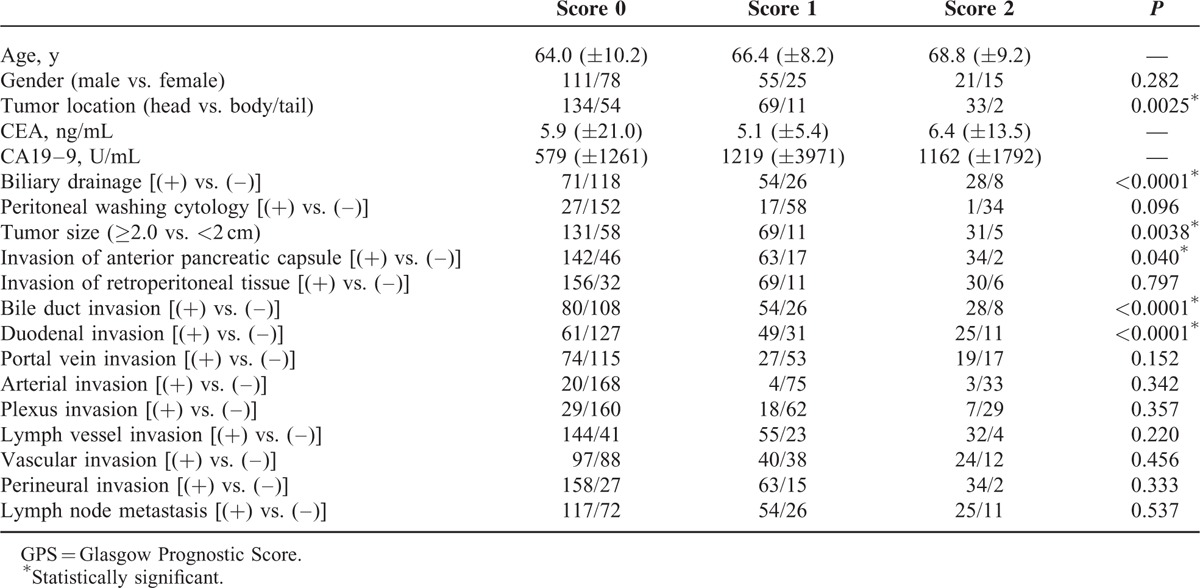
Correlation Between GPS Score and Clinicopathological Factors

## DISCUSSION

It is widely recognized that survival outcome of cancer patients is not merely dependent on tumor characteristics itself but also on various host-related factors that could play a critical role. In particular, so-called cancer-associated inflammation is a key determinant of cancer initiation, progression, metastasis, and survival.^7,20^ That is, the tumor can trigger regional inflammatory responses, resulting in the release of pro-inflammatory cytokines around the tumor, which could lead to the formation of an inflammatory microenvironment.^7,8^ Several gastroenterological cancers are well known to be profoundly associated with inflammation such as those caused by the hepatitis B virus^21^ or *Helicobacter pylori*^22^ infections, for example, pancreatic cancer. In the current study, we explored clinically useful prognostic parameters, including host and tumor-related parameters in patients with resected pancreatic cancer. Hence, the clinical value of the GPS, mGPS, NLR, PLR, PI, and PNI as recognized inflammation-based prognostic scores was compared in our large cohort. To the best of our knowledge, the current study is the first to extensively explore the prognostic value of various inflammation-based prognostic scores as predictors of prognosis in pancreatic ductal adenocarcinoma after curative surgery.

Since Forrest et al^23^ first reported their study of inoperable nonsmall cell lung cancer, the GPS has been shown to be a favorable predictor of survival in patients with various cancers. CRP and albumin as acute-phase proteins, which constitute the GPS score, are sensitive and reliable markers that reflect the systemic-inflammatory response in cancer patients. The mGPS was subsequently established as a prognostic marker, which has been evaluated and validated in both operable and inoperable cancers.^24^ Recently, a Glasgow Inflammation Outcome Study, conducted by Proctor et al,^25^ revealed that mGPS had a prognostic value in cancer independent of the tumor site and was superior to other inflammation-based prognostic scores in terms of differentiating good from poor prognostic groups. When patients were classified on the basis of their GPS or mGPS score, the survival curve in each group was clearly stratified, which was more remarkable in GPS than in mGPS. Recently, the other prognostic scores (NLR, PLR, PI, and PNI) have also been actively studied in pancreatic cancer;^9,26–29^ but our data have shown no difference in survival when patients were stratified according to these scores. Furthermore, multivariate analysis revealed that lymph node metastasis, positive peritoneal washing cytology, and the GPS score were significant prognostic factors. Therefore, our study demonstrated that the GPS could be an independent host-related prognostic marker in patients with resected pancreatic cancer.

Interestingly, when the correlation between the GPS score and clinicopathological factors was analyzed, factors that were statistically significant included location of the tumor in the head of the pancreas, larger tumor size, bile duct invasion, and duodenal invasion. In general, larger tumors in the head of the pancreas were often accompanied by bile duct or duodenal invasion, resulting in biliary obstruction and infection. Consequently, systemic inflammation and malnutrition could be caused in such a situation, which might be a rationale for why the GPS could be the most significant inflammation-based prognostic score in pancreatic cancer.

Although the detailed pathogenesis is still unclear, a marked systemic-inflammatory response is certainly associated with patients’ nutritional, functional, and immunological decline. We have reported that ensuring sufficient preoperative control of infection by biliary drainage is one of the most important considerations in the management of pancreatic resection with regard to the decrease in postoperative complications (e.g., surgical site infection and pancreatic fistula).^30^ Prognostic significance of GPS indicates, in addition, that preoperative management of biliary infection could improve survival outcomes. Related to the issue of the relationship between infection and cancer, there were some studies regarding the effect of aspirin and other nonsteroidal anti-inflammatory drugs on the prevention of esophagus, stomach, and colon cancer,^31–33^ and prevention of the systemic inflammatory response through, for example, interleukin (IL)-6, could be a potential therapeutic target in an attempt to dampen the exaggerated systemic-inflammatory response.^34^

Prognostic significance of GPS suggests relevance of infection control and nutritional support before surgery and could support our concept of rigorous preoperative biliary drainage. It could in addition provide a rationale for preoperative nutritional support, possibly utilizing oral nutritional supplement. On the contrary, GPS could be reflecting biology of the tumor itself, in which case, besides considering GPS as a mere prognostic marker, it could be used to select patients who are indicated for surgery-first strategy for preoperative treatments from the viewpoint of both controlling the tumor before surgery and avoiding futile surgery in case of rapid cancer progression.

A potential limitation of this study is that it was a retrospective, single-center study. Therefore, a large-scale prospective validation study is needed to confirm the results. Second, patients who had the tumor in the head of the pancreas often underwent biliary drainage procedures during the preoperative period, which may represent a potential confounding factor. Because CRP, white blood cells, and neutrophils are markers of acute inflammation, they could have been elevated or modified secondary to acute infection and subsequent biliary drainage.

In conclusion, our study demonstrated that the GPS, an inflammation-based prognostic score, was superior to the other inflammation-based prognostic scores and therefore, could be an independent predictive marker in patients with resected pancreatic cancer.

## Supplementary Material

Supplemental Digital Content

## References

[1] Pawlik TM Gleisner AL Cameron JL Prognostic relevance of lymph node ratio following pancreaticoduodenectomy for pancreatic cancer. *Surgery* 2007; 141:610–618. 17462460 10.1016/j.surg.2006.12.013

[2] Fortner JG Klimstra DS Senie RT Tumor size is the primary prognosticator for pancreatic cancer after regional pancreatectomy. *Ann Surg* 1996; 223:147–153. 8597508 10.1097/00000658-199602000-00006 PMC1235090

[3] Fujii T Nakao A Yamada S Vein resections >3 cm during pancreatectomy are associated with poor 1-year patency rates. *Surgery* 2015; 157:708–715. 25704426 10.1016/j.surg.2014.12.002

[4] Neoptolemos JP Stocken DD Dunn JA Influence of resection margins on survival for patients with pancreatic cancer treated by adjuvant chemoradiation and/or chemotherapy in the ESPAC-1 randomized controlled trial. *Ann Surg* 2001; 234:758–768. 11729382 10.1097/00000658-200112000-00007 PMC1422135

[5] Raut CP Tseng JF Sun CC Impact of resection status on pattern of failure and survival after pancreaticoduodenectomy for pancreatic adenocarcinoma. *Ann Surg* 2007; 246:52–60. 17592291 10.1097/01.sla.0000259391.84304.2b PMC1899216

[6] Yamada S Fujii T Sugimoto H Aggressive surgery for borderline resectable pancreatic cancer: evaluation of National Comprehensive Cancer Network guidelines. *Pancreas* 2013; 42:1004–1010. 23532000 10.1097/MPA.0b013e31827b2d7c

[7] Mantovani A Allavena P Sica A Cancer-related inflammation. *Nature* 2008; 454:436–444. 18650914 10.1038/nature07205

[8] Grivennikov SI Greten FR Karin M Immunity, inflammation, and cancer. *Cell* 2010; 140:883–899. 20303878 10.1016/j.cell.2010.01.025 PMC2866629

[9] Martin HL Ohara K Kiberu A Prognostic value of systemic inflammation-based markers in advanced pancreatic cancer. *Intern Med J* 2014; 44:676–682. 24750233 10.1111/imj.12453

[10] McKay CJ Glen P McMillan DC Chronic inflammation and pancreatic cancer. *Best Prac Res Clin Gastroenterol* 2008; 22:65–73. 10.1016/j.bpg.2007.11.007 18206813

[11] Forrest LM McMillan DC McArdle CS A prospective longitudinal study of performance status, an inflammation-based score (GPS) and survival in patients with inoperable non-small-cell lung cancer. *Br J Cancer* 2005; 92:1834–1836. 15870712 10.1038/sj.bjc.6602591 PMC2361776

[12] Crumley AB McMillan DC McKernan M Evaluation of an inflammation-based prognostic score in patients with inoperable gastro-oesophageal cancer. *Br J Cancer* 2006; 94:637–641. 16479253 10.1038/sj.bjc.6602998 PMC2361199

[13] McMillan DC Canna K McArdle CS Systemic inflammatory response predicts survival following curative resection of colorectal cancer. *Br J Surg* 2003; 90:215–219. 12555298 10.1002/bjs.4038

[14] Erlinger TP Platz EA Rifai N C-reactive protein and the risk of incident colorectal cancer. *JAMA* 2004; 291:585–590. 14762037 10.1001/jama.291.5.585

[15] Ramsey S Lamb GW Aitchison M Evaluation of an inflammation-based prognostic score in patients with metastatic renal cancer. *Cancer* 2007; 109:205–212. 17149754 10.1002/cncr.22400

[16] Yamamura K Sugimoto H Kanda M Comparison of inflammation-based prognostic scores as predictors of tumor recurrence in patients with hepatocellular carcinoma after curative resection. *J Hepatobiliary Pancreat Sci* 2014; 21:682–688. 24823966 10.1002/jhbp.114

[17] Nakao A Takagi H Isolated pancreatectomy for pancreatic head carcinoma using catheter bypass of the portal vein. *Hepatogastroenterology* 1993; 40:426–429. 8270230

[18] Nakao A Takeda S Inoue S Indications and techniques of extended resection for pancreatic cancer. *World J Surg* 2006; 30:976–982. 16736324 10.1007/s00268-005-0438-6

[19] International Union Against Cancer. TNM Classification of Malignant Tumors. 7th ed. New York: Wiley-Blackwell; 2009.

[20] Hanahan D Weinberg RA Hallmarks of cancer: the next generation. *Cell* 2011; 144:646–674. 21376230 10.1016/j.cell.2011.02.013

[21] Wang DS Chen DL Ren C ABO blood group, hepatitis B viral infection and risk of pancreatic cancer. *Int J Cancer* 2012; 131:461–468. 21858814 10.1002/ijc.26376

[22] Trikudanathan G Philip A Dasanu CA Association between Helicobacter pylori infection and pancreatic cancer. A cumulative meta-analysis. *JOP* 2011; 12:26–31. 21206097

[23] Forrest LM McMillan DC McArdle CS Comparison of an inflammation-based prognostic score (GPS) with performance status (ECOG) in patients receiving platinum-based chemotherapy for inoperable non-small-cell lung cancer. *Br J Cancer* 2004; 90:1704–1706. 15150622 10.1038/sj.bjc.6601789 PMC2409737

[24] McMillan DC The systemic inflammation-based Glasgow Prognostic Score: a decade of experience in patients with cancer. *Cancer Treat Rev* 2013; 39:534–540. 22995477 10.1016/j.ctrv.2012.08.003

[25] Proctor MJ Morrison DS Talwar D A comparison of inflammation-based prognostic scores in patients with cancer. A Glasgow Inflammation Outcome Study. *Eur J Cancer* 2011; 47:2633–2641. 21724383 10.1016/j.ejca.2011.03.028

[26] Smith RA Bosonnet L Raraty M Preoperative platelet-lymphocyte ratio is an independent significant prognostic marker in resected pancreatic ductal adenocarcinoma. *Am J Surg* 2009; 197:466–472. 18639229 10.1016/j.amjsurg.2007.12.057

[27] Bhatti I Peacock O Lloyd G Preoperative hematologic markers as independent predictors of prognosis in resected pancreatic ductal adenocarcinoma: neutrophil-lymphocyte versus platelet-lymphocyte ratio. *Am J Surg* 2010; 200:197–203. 20122680 10.1016/j.amjsurg.2009.08.041

[28] Garcea G Ladwa N Neal CP Preoperative neutrophil-to-lymphocyte ratio (NLR) is associated with reduced disease-free survival following curative resection of pancreatic adenocarcinoma. *World J Surg* 2011; 35:868–872. 21312035 10.1007/s00268-011-0984-z

[29] Wang DS Luo HY Qiu MZ Comparison of the prognostic values of various inflammation based factors in patients with pancreatic cancer. *Med Oncol* 2012; 29:3092–3100. 22476808 10.1007/s12032-012-0226-8

[30] Fujii T Yamada S Suenaga M Preoperative internal biliary drainage increases the risk of bile juice infection and pancreatic fistula after pancreatoduodenectomy: a prospective observational study. *Pancreas* 2015; 44:465–470. 25423556 10.1097/MPA.0000000000000265

[31] Baron JA Sandler RS Nonsteroidal anti-inflammatory drugs and cancer prevention. *Annu Rev Med* 2000; 51:511–523. 10774479 10.1146/annurev.med.51.1.511

[32] Langman MJ Cheng KK Gilman EA Effect of anti-inflammatory drugs on overall risk of common cancer: case-control study in general practice research database. *BMJ* 2000; 320:1642–1646. 10856067 10.1136/bmj.320.7250.1642 PMC27410

[33] Garcia-Rodriguez LA Huerta-Alvarez C Reduced risk of colorectal cancer among long-term users of aspirin and nonaspirin nonsteroidal antiinflammatory drugs. *Epidemiology* 2001; 12:88–93. 11138826 10.1097/00001648-200101000-00015

[34] Yamada S Okumura N Wei L Epithelial to mesenchymal transition is associated with shorter disease-free survival in hepatocellular carcinoma. *Ann Surg Oncol* 2014; 21:3882–3890. 24833103 10.1245/s10434-014-3779-2

