# Supplementary material for: Clinical Implication of Inflammation-Based Prognostic Score in Pancreatic Cancer: Glasgow Prognostic Score Is the Most Reliable Parameter
Source: Medicine (Baltimore). 2016 May 6;95(18):e3582. doi: 10.1097/MD.0000000000003582 (PMC4863804; doi:10.1097/MD.0000000000003582)
Supplement: Supplemental Digital Content [file medi-95-e3582-s001.pdf]

Supplementary TABLE 1. Inflammation-based prognostic score

Scoring system	Score
**Glasgow prognostic score (GPS)**	
CRP (≤1.0 mg/l) and albumin (≥3.5 g/dl)	0
CRP (≤1.0 mg/l) and albumin (<3.5 g/dl)	1
CRP (>1.0 mg/l) and albumin (≥3.5 g/dl)	1
CRP (>1.0 mg/l) and albumin (<3.5 g/dl)	2
**modified GPS**	
CRP (≤1.0 mg/l) and albumin (≥3.5 g/dl)	0
CRP (≤1.0 mg/l) and albumin (<3.5 g/dl)	0
CRP (>1.0 mg/l)	1
CRP (>1.0 mg/l) and albumin (<3.5 g/dl)	2
**Nentrophil lymphocyte ratio (NLR)**	
Neutrophil count:lymphocyte count <3:1	0
Neutrophil count:lymphocyte count ≥3:1	1
**Platelet lymphocyte ratio (PLR)**	
Platelet count:lymphocyte count <150:1	0
Platelet count:lymphocyte count ≥150:1	1
**Prognostic index (PI)**	
CRP (≤1.0 mg/l) and white cell count (≤11,000/µl)	0
CRP (≤1.0 mg/l) and white cell count (>11,000/µl)	1
CRP (>1.0 mg/l) and white cell count (≤11,000/µl)	1
CRP (>1.0 mg/l) and white cell count (>11,000/µl)	2
**Prognostic nutritional index (PNI)**	
10 × Albumin (g/dl) + 0.005 × total lymphocyte count (/µl) ≥45	0
10 × Albumin (g/dl) + 0.005 × total lymphocyte count (/µl) <45	1

CRP; C-reactive prote
